# Comparing AI chat tools and PubMed for medical literature searches

**DOI:** 10.5195/jmla.2026.2284

**Published:** 2026-10-01

**Authors:** A'Llyn Ettien, Kathleen Downing, Rebecca Drouhard, Bridget Gunn, Megan McNichol, Kate Silfen, Allie Tatarian

**Affiliations:** 1 aettien3@bu.edu, Head of Resource Sharing and Discovery, Alumni Medical Library, Boston University, Boston, MA; 2 downing.kathleen@gmail.com, Retired, formerly Digital Library Coordinator, Network of the National Library of Medicine Public Health Coordination Office, University of Massachusetts Chan Medical School, Worcester, MA; 3 Rebecca.Drouhard@umassmed.edu, Librarian, Library Education and Clinical Services, Lamar Soutter Library, UMass Chan Medical School, Worcester, MA; 4 bridget.gunn@baystatehealth.org, Information and Knowledge Services Librarian, Health Sciences Library, Baystate Health, Springfield, MA; 5 mmcnich3@bilh.org, Manager, Agoos Medical Library & Information Commons, Beth Israel Deaconess Medical Center, Boston, MA; 6 ksilfen@bu.edu, Health Sciences Librarian, Boston University Libraries, Boston University, Boston, MA; 7 allie.tatarian@tufts.edu, Research & Instruction and Data Librarian, Hirsh Health Sciences Library, Tufts University, Boston, MA

**Keywords:** AI, literature searches, comparison

## Abstract

**Objective:**

This investigation compared six AI chat tools (ChatGPT, Claude, Copilot, Gemini, OpenEvidence, and Perplexity) to each other and to PubMed to assess their utility in medical literature searches.

**Methods:**

Each tool was prompted with two standardized prompts simulating an academic and a clinical literature need. Prompts were repeated three times per tool, and results recorded for each prompt iteration.

**Results:**

Main findings include the range in number of real and hallucinated articles for each AI tool, from no hallucinations in OpenEvidence to more than 80% hallucinated citations in ChatGPT. Across all tools, most real articles were assessed as relevant. Continued prompting of all tools produced duplicate results. Most tools showed very little consistency in citations provided to different researchers, suggesting low probability of replicating search results.

**Conclusions:**

Though limited by small sample size, inconsistent data, and the rapid pace of change in LLM technology, this study suggests some advantages and drawbacks of using AI tools for literature searching compared with PubMed. AI tools with few or no hallucinations and high relevance, such as OpenEvidence and Perplexity, performed well in terms of simply supplying a few article citations on the topic, but small results sets and lack of consistency and transparency limit their value for thorough research. These results suggest that AI tools are not presently a viable replacement for PubMed or other academic databases for systematic reviews or other in-depth literature searches but may be adequate for limited information needs or as a complement to database or web searches.

## INTRODUCTION

Since the 2022 release of widely available large language model (LLM) chatbot tools, there has been interest in how this type of artificial intelligence (AI) might assist scientific research and writing. Numerous studies examine AI ability to formulate search queries, summarize data and articles, improve written work, and locate relevant literature citations [1–5]. Xu et al. found a majority of medical students reported using LLM tools in some way [6], showing this topic’s relevance to medical libraries. Concerns with AI usage include ‘hallucinations’ (users relying on inaccurate information/citing nonexistent articles; frustration and lost time for library users and staff attempting to locate nonexistent articles in reference consultations or ILL requests), and opaque retrieval mechanisms that may provide inadequate or irrelevant results for literature search prompts. These tools have generally been found to perform better at summarizing and writing than at listing specific articles or information sources [7–13].

A literature search identified other studies investigating AI tools’ provision of article citations. Several authors noted that ChatGPT can hallucinate large numbers of citations [7,8,12–14]. Other studies compared citations produced by ChatGPT and Google Bard (now Gemini), concluding that both generated significant numbers of hallucinations [9,10,15]. Van der Werf [16], leading a workshop evaluating the citation-producing capabilities of Perplexity, Elicit, and SciSpace, reported participants found these tools fast and easy to use, but not transparent or reliable.

Previous studies clearly identify hallucination (both citations and information) as a concern with LLM tools, indicating that plausibly titled hallucinated works and the ‘confidence’ of tools presenting citations and summarized information makes it difficult for users to determine whether information is accurate [8,9]. Inability of LLMs to accurately discover known articles is found in multiple studies, along with inaccurate summarization [17–19]. The number of previous studies examining ChatGPT highlight its importance as the best-known AI chatbot, familiar to large numbers of users although not a scholarly tool. This paper builds on earlier works, comparing six AI tools to PubMed and providing data on hallucination rates, provision of relevant citations, and consistency and duplication of results among participants. The current study attempted to approximate and evaluate the experience of a non-expert searcher requesting medical literature from several LLM tools. We were interested in how well these tools performed this task at a basic level, producing real articles relevant to the topic of interest; we did not assess accuracy or recency of information in the articles provided. While article quality is obviously a critical component of how well these tools perform on literature searches, it was outside the scope of this project.

## METHODS

### Study Design and Participants

This original investigation was conducted by 13 health sciences librarians representing 10 Massachusetts institutions; depending on availability and access, 4–7 librarians tested each tool. The study was designed to evaluate performance of six AI chat tools in retrieving peer-reviewed literature in response to structured prompts. While the generative AI field contains tools specifically for academic writing, (i.e. Research Rabbit, SciSpace, Elicit), we were interested in the experience of users relying on well-known general purpose tools. Previous studies of AI and medical students suggest these may be the tools most users rely on [11,20,21], and familiarity with a tool for one purpose (e.g. summary of a topic) may logically facilitate its use for other purposes (e.g., searching for relevant literature). We did not consider tools requiring paid accounts, though some offer more access with paid versions, or, in the case of OpenEvidence, are limited to healthcare providers and possibly inaccessible to some users.

We used PubMed as a comparison since, as a free resource, PubMed is the clearest counterpart to free AI tools, as well as the best-known medical literature database in the US.

Tools evaluated included ChatGPT, Claude, Copilot (formerly Bing AI), Gemini (formerly Bard), OpenEvidence, and Perplexity AI. Terminology was not established in advance, and participants reported chatbot versions inconsistently. Of 7 ChatGPT users, 2 reported 4o Mini, 1 4o Mini/’auto’, 1 ‘auto’, 2 unspecified, and 1 unspecified/‘free’. Of 5 Claude users, 1 reported paid version, 2 reported 3.5, and 2 indicated ‘free’/unspecified. Copilot at that time used OpenAI’s ChatGPT model, so of 8 Copilot users, 1 reported Creative ChatGPT 4o Mini,/ChatGPT4, 3 reported Creative Chat GPT4, 2 reported Creative, 1 Creative/unspecified, and 1 Balanced. Six users of Gemini and 8 each of OpenEvidence and Perplexity all indicated ‘free’ or unspecified versions. Though inconsistency of versions used/reported, and of prompters per tool, limited data standardization, we chose to use what we had rather than discard input. Since our goal was not to provide definitive information on any specific product, but rather to gain insight into the basic experience of using these tools for literature searching, we accepted a variety of versions, incorporating whatever tool variant was accessible to a particular user. While this limits what can be decisively said about any individual version (many of which have since been superseded), it allows us to examine more broadly the overall experience of using LLM tools for literature searches.

### Research Questions

The team developed five questions for investigation:

1. For each tool, are the recommended article citations real or hallucinated?
2. For each tool, if the article is real, is it relevant to the prompt topic?
3. For each tool, how many times can we use the same prompt before duplication and/or hallucination occur?
4. Do multiple simultaneous users of the same tool get the same citations in their results?
5. For each tool, how do the results compare to the results from a PubMed search for the same topic?

### Prompts

Two primary prompts were developed, simulating educational and clinical information needs.

- **Educational Prompt**: “Please provide peer-reviewed articles about Teach-Back for patient education at discharge.”
- **Clinical Prompt**: “Please provide real peer-reviewed articles in APA format about the Neutropenic diet, low bacterial diet, or low microbial diets as they relate to sepsis or length of stay.”

Each prompt was followed by three iterations requesting more articles on the topic with the word “additional” (i.e. “Please provide additional peer-reviewed articles about Teach-Back for patient education at discharge”) making four total interactions per tool, per prompt. The exception, OpenEvidence, allowed only two follow-ups at the free level, making three total interactions per prompt.

### Data Collection

Data collection was organized through email and Zoom meetings. Participating librarians submitted prompts to each AI chat tool and recorded responses. The first (educational) prompt was used in a group session Aug. 21, 2024; the second (clinical) prompt was used Sept. 18, 2024. Participants unable to attend the Aug. 21 session completed prompts and sent results over the next week (on or before Aug. 28, 2024).

For database comparison, we ran simple PubMed searches using the queries ***(teach-back) AND (patient education) AND (discharge)***, date limit Aug. 21, 2024, and ***(neutropenic diet OR low bacterial diet OR low microbial diet) AND (sepsis OR length of stay)***, date limit Sept. 18, 2024. While our professional inclination would normally be more sophisticated search strategies, our goal was to retrieve results similar to those a non-expert PubMed user might receive. We felt this both a fairer comparison to our non-expert prompt texts, and more relevant to an average literature seeker’s experience.

### Evaluation Criteria for Each Research Question

- **Hallucinations** were defined as citations we could not verify. Researchers attempted to verify each unique citation a chatbot provided by searching PubMed, the journal if listed, and via web search. For purposes of analysis we counted as ‘real’ citations that we scored as ‘correct’ (fully matching an existing source) or ‘partially correct’ (mostly matching an existing source, but some missing/incorrect information, i.e. publication date or volume number). Any citation for which we found no matching source was considered a hallucination for the tool providing it. Total hallucinations for each tool were tallied. (Our second prompt requested articles “in APA format” hoping this would produce more complete citations, but we did not evaluate results on adherence to formatting guidelines: we counted as “real” any citation for which we found a close/complete match, regardless of its formatting in APA or another style. We also counted as “real” actual citations that were not peer-reviewed articles, e.g. dissertations, book chapters, and web pages.)
- **Relevance** was assessed qualitatively based on alignment with prompt topic. Each unique non-hallucinated citation was reviewed at the title level and, if needed, abstract level, by two researchers; a third was tie-breaker. Each citation was considered a relevant or irrelevant result for the tool providing it. Total relevant/irrelevant results were tallied. Rulings were based on topic, not format: a non-peer-reviewed article could be relevant, though not fully meeting prompt criteria. Number Needed to Read (number of results screened / number of results deemed relevant) was also calculated for each tool using the total unique citations it provided.
- **Duplication** was tracked for each tool, considering unique citations received in response to the initial prompt (P1) and three follow-ups (P2–P4). We defined duplication as any prompter seeing a specific citation for the second+ time. Each citation was marked either ‘duplicated’ or ‘new’ in each prompt response. Total duplicated citations per iteration were tallied. Number of prompters seeing a citation again was not counted (2 people seeing 1 citation the second time each = 1 duplicated citation in that response), but repeated duplication over multiple responses was (1 citation provided in 3 total responses = 2 duplications total).
- **Consistency** was calculated for each tool using formula (([number of unique views of non-hallucinated citations] / [number of non-hallucinated citations] / [number of prompters in session]) * 100). A 100 score indicates complete consistency (all prompters seeing all citations): smaller numbers indicate less consistency (less likelihood of multiple people seeing a citation). This “consistency score” for each tool expresses the percent chance of any given prompter seeing any given citation that tool provided. Since number of prompters in each session varied, the consistency score’s lower limit also varied, smaller in larger groups. This variability, as well as the variation in tool versions noted above, makes our consistency score a rough value: it should not be interpreted as a precise measurement.

**Comparison to PubMed** involved evaluating PubMed results for topic relevance following the same process used for chatbot results. All PubMed results were real. Searches were not repeated, so duplication was not considered (but would have been complete). Consistency will be 100 for anyone using these search strategies.

### Data Analysis

Data was compiled and analyzed using Microsoft Excel to calculate total numbers and mean. Results were further analyzed using 2-factor ANOVA with replication, examining the performance of tools between the two prompts, with tool name and prompt type (‘Teach-back’ or ‘Neutropenia’) as factors. Proportion of real/non-hallucinated citations and relevant citations were analyzed as dependent variables. Duplication/repetition was assessed using 2-factor ANOVA with replication, with tool name and duplication over prompt iterations P2-4. Since OpenEvidence only allowed three total repeats of each prompt, for all ANOVA calculations we used the mean of the existing responses to impute data for a fourth prompt. Though conclusions are necessarily limited given our small sample sizes and inconsistent data, results are reported below.

## RESULTS

### Overall Trends and Behaviors

Every AI chatbot except OpenEvidence produced hallucinated article citations. Hallucinated citations could appear with any prompt in tools producing them, commonly in response to Prompt 1.

Every chatbot repeated real article citations, sometimes within the same response. Results could repeat both between prompt iterations for the same prompter (duplication), and between different prompters (consistency). Hallucinated citations were usually not repeated, but were occasionally duplicated between responses to one prompter, or, rarely, to different prompters.

We did not attempt to assess whether tools provided accurate information if asked to summarize the literature regarding our prompt topics, and while in some cases they provided a summary even though it was not requested, we did not include or evaluate this content. It may be that hallucination-prone tools could respond adequately to summary requests even while unable to “cite their sources” when asked for articles, but this determination was outside the project scope.

Any prompter using any AI chatbot could expect on average 4.9 article citations (including duplicate and hallucinated results) in response to any one prompt. Most chatbots gave similar numbers. The exception, Perplexity, averaged 7.5 citations/prompt. Excluding Perplexity, the average number of citations/prompt was 4.3.

### Real and Hallucinated Articles

Five of six AI tools included hallucinated citations in results (see Table 1). Only OpenEvidence provided entirely real citations, though Perplexity returned few hallucinations (10/334, 3.0%). ChatGPT’s results were mostly hallucination (125/177, 70%), followed by Gemini (97/153, 63%). Copilot’s citations were 42% hallucinated (84/202), and Claude’s 27% (37/138). Deduplication changes the numbers slightly. Considering only unique citations, ChatGPT still produced most hallucinations, with 125/154 article citations (81%) hallucinated. Perplexity remains lowest after OpenEvidence, with 3/46 (6.5%) hallucinated. Copilot’s citations were 73% hallucinations (72/99), Gemini’s 72% (63/88), and Claude’s 40% (31/78).

**Table 1:** Number of real (R) and hallucinated (H) citations retrieved from each tool for each iteration of two prompts. Includes irrelevant results, repetition of unique results to multiple prompters, and duplication of results to individual prompters.

Educational Prompt (“Teach-back”)											
Tool	No. of Prompters	*Prompt 1*		*Prompt 2*		*Prompt 3*		*Prompt 4*		Total	
		*R*	*H*	*R*	*H*	*R*	*H*	*R*	*H*	*R*	*H*
ChatGPT	6	3	20	6	19	6	22	4	21	19	82
Claude	4	8	7	12	6	8	8	11	7	39	28
Copilot	7	30	0	17	18	14	11	15	15	76	44
Gemini	5	16	3	15	14	7	20	9	20	47	57
OpenEvidence	6	15	0	49	0	30	0	--	--	94	0
Perplexity	6	33	0	53	2	37	0	38	1	161	3
PubMed	1	36	--	--	--	--	--	--	--	36	--

Clinical Prompt (“Neutropenic diet”)											
Tool	No. of Prompters	*Prompt 1*		*Prompt 2*		*Prompt 3*		*Prompt 4*		Total	
		*R*	*H*	*R*	*H*	*R*	*H*	*R*	*H*	*R*	*H*
ChatGPT	5	7	10	9	10	10	11	7	12	33	43
Claude	4	15	0	20	1	17	3	10	5	62	9
Copilot	5	8	12	8	12	13	8	13	8	42	40
Gemini	4	2	10	4	15	2	12	1	9	9	46
OpenEvidence	5	20	0	23	0	26	0	--	--	69	0
Perplexity	5	43	1	42	1	40	2	38	3	163	7
PubMed	1	98	--	--	--	--	--	--	--	98	--

Two-way ANOVA showed a significant difference in the number of real results between the tools (F(5,36)=43.7, p<0.001), indicating some tools were more likely than others to provide real results, and a barely significant interaction between tools and prompts (F(5,36)=2.57, p=0.04), suggesting that some tools were more likely to provide real results for one of the two prompts. There was no significant difference in the proportion of real results between the two prompts (F(5,36)=1.28, p=0.26), suggesting that neither the clinical nor educational prompt was overall more likely to retrieve real results.

For fabricated results, ANOVA showed a significant difference in the proportion of hallucinations between the tools (F(4,30)=13.53, p<0.001), and between the two prompts (F(4,30)=7.5, p=0.01) suggesting that some tools were more likely to hallucinate overall, and that one of the two prompts was more likely to retrieve hallucinated results. There was no significant interaction between tools and prompts (F(4,30)=1.19, p=0.33), suggesting that no particular tool provided more hallucinations in response to either prompt.

These findings support the observations of our raw data in showing that some tools provided more real or hallucinated results than others, and may also indicate that the text of one of our prompts was less likely to receive hallucinated results in general, and more likely to receive real results from certain tools. Given the minimal differences in phrasing, it is unclear whether tools respond differently to requests for educational than for clinical literature, or whether adding “real” and/or “APA format” (as in our second, clinical prompt) may in fact be somewhat effective.

### Relevant Citations

For citations determined to be real, the majority were relevant to the topic (see Figure 1). Overall relevance ranged from 78% (Copilot) to 100% (OpenEvidence): most tools returned at least 90% relevant citations. Hallucinated citations uniformly appeared relevant based on title, but, since articles did not exist, could not provide information relevant to the prompt.

**Figure 1:**
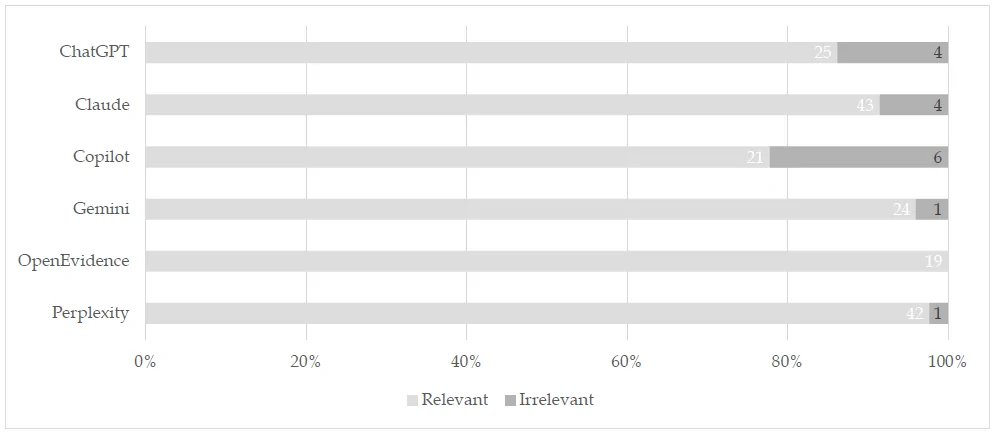
Number of unique relevant and irrelevant citations for each AI tool (clinical and educational prompts combined). Duplicates and hallucinated citations excluded.

We assessed relevance only for real citations, but researchers making LLM tools part of formal search strategy might consider “screening” as a process that includes eliminating both hallucinatory and irrelevant real works from results. In this case, an established metric like Number Needed to Read (NNR) is useful. We calculated NNR for each tool using the total unique citations it provided (see Table 2). Even including hallucinations, all scored well, likely because our searches were fairly specific and our relevancy criteria broad.

**Table 2:** NNR for each tool

ChatGPT	Claude	Copilot	Gemini	OpenEvidence	Perplexity	PubMed
6.16	1.81	4.71	3.66	1	1.09	3.26

ANOVA testing showed a significant difference in the number of *relevant* results returned by each tool (F(5,36)=46.66, p<0.001) as well as a significant interaction between the tools and the two prompts (F(5,36)=2.92, p=0.02). There was no significant overall difference in the number of relevant results between the two prompts (F(5,36)=2.59, p=0.11). This suggests that some tools are more likely to provide relevant results than others, particularly in response to one of the prompts, but that neither prompt was more likely to receive relevant results overall.

There were also significant differences in number of *irrelevant* results (F(5,36)=4.80, p=0.001), number of irrelevant results per prompt (F(5,36)=8.10, p=0.007), and interaction between tools and prompts (F(5,36)=3.99, p=0.005), suggesting that some tools are more likely to provide irrelevant results (unsurprising given that some were more likely to provide hallucinations, which cannot be relevant), and that prompt text may matter when it comes to relevance (again perhaps linked to number of hallucinations).

### Duplication and Hallucination when Repeating Prompts

Duplication of citations to individual prompters occurred for each AI tool (Figure 2), with some tools repeating a citation in the initial response. Hallucinations appeared in every AI tool except OpenEvidence and occurred with each prompt (Figure 3).

**Figure 2:**
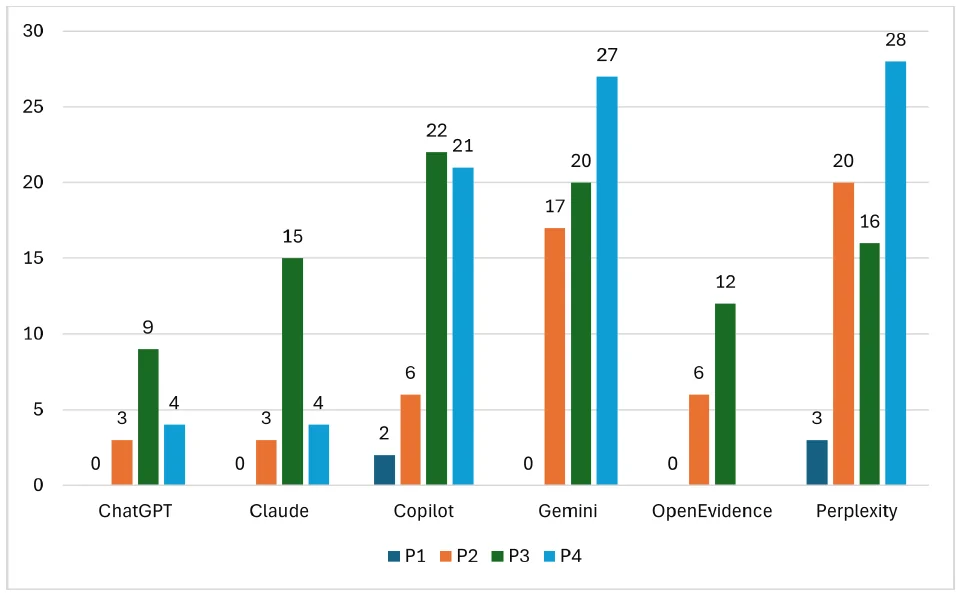
Times unique citations repeated to individual prompters and for which prompt iteration(s) repetition occurred. Clinical and educational prompts combined.

Note: OpenEvidence was prompted three times (P1-P3).

**Figure 3:**
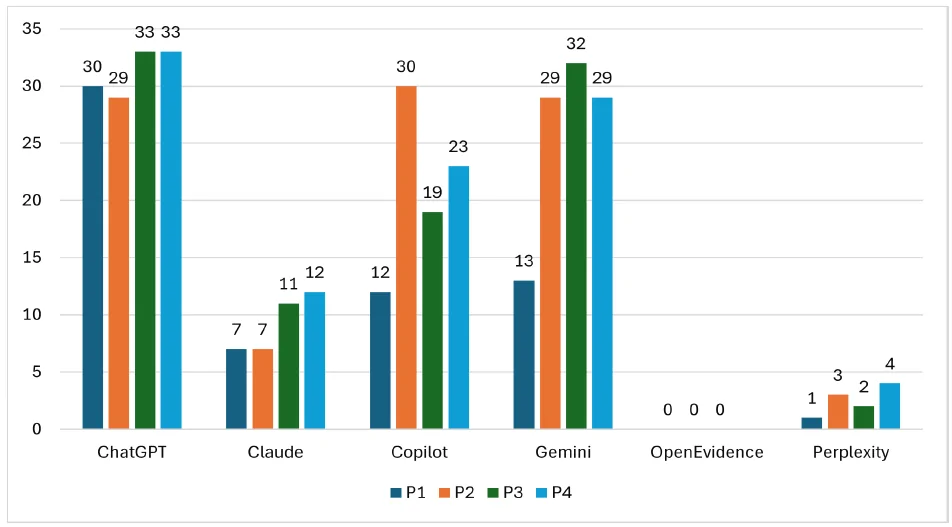
Number of hallucinated citations per prompt iteration. Clinical and educational prompts combined.

Note: OpenEvidence was prompted three times (P1-P3).

### Repetition of Results when Repeating Prompts

ANOVA testing showed no difference between tools (F(5,24)=1.53, p=0.21) or between the numbers of new/repeated citations over the course of multiple iterations of the prompt (F(5,24)=2.13, p=0.15), and no interaction between the variables (F(5,24)=1.71, p=0.16) for the first, educational prompt. For the second, clinical prompt we did find a significant difference between tools (F(5,24)=3.44, p=0.01), as well as a significant interaction between tools and new/repeated citations (F(5,24)=4.33, p=0.005), but there was no significant difference in the overall number of new and repeated results (F(5,24)=0.65, p=0.42). This may suggest that while some tools may be more likely to provide new or repeated results generally, at least in response to one of the two prompts, each prompt iteration was, overall, as likely to retrieve a repeated result as a new one. We repeated prompts verbatim aside from the word “additional;” it is possible tools would have provided more new articles if we had modified prompts each time while maintaining key components of the topic, as we would do with databases or on the web.

### Consistency of Results for AI Tools

OpenEvidence demonstrated 100% consistency with the clinical prompt (all six articles were retrieved by all five searchers); for the educational prompt, consistency was 73%. No other tool showed complete consistency for either prompt. Consistency scores for combined prompts: OpenEvidence 87; Perplexity 63; Claude 50; Copilot 32; Gemini 24; ChatGPT 21.

### Comparing AI and PubMed Results

PubMed retrieved 134 results for both topics combined (36 educational, 98 clinical); 41 were deemed relevant, slightly below Claude’s 43 and Perplexity’s 42 (see Figure 4). PubMed retrieved 93 irrelevant results, for a relevancy score of 31%, lower than any AI tool (Figure 1). PubMed’s NNR fell between tools with fewer and those with more hallucinated results, suggesting that high hallucination rates may be analogous to high irrelevancy rates from the user perspective: both mean sifting through unusable results. PubMed retrieved considerably more results per query than the chatbots. While Perplexity and PubMed produced almost the same number of real, relevant citations (see Figure 4), and Perplexity included far fewer irrelevant citations, it took 44 total prompts (5 and 6 prompters each repeating prompts 4 times) to retrieve Perplexity’s 42 relevant results, while 2 searches retrieved PubMed’s 41 relevant results. This may be either an advantage or disadvantage depending on user need: retrieving many articles at once facilitates a broader view of available literature on a topic and more efficient discovery of multiple articles for a thorough search, but high retrieval of irrelevant articles may be frustrating for users with more limited information needs.

**Figure 4:**
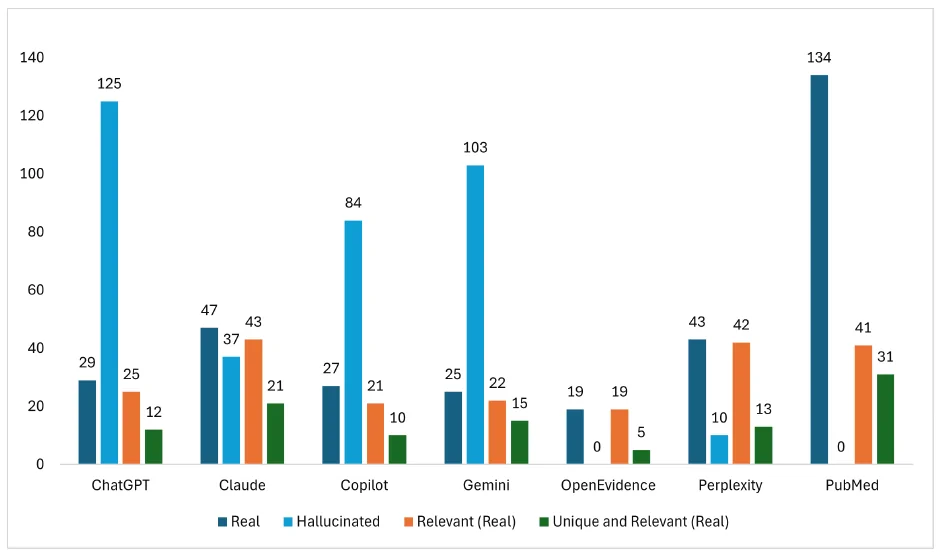
Total real, hallucinated, relevant, and unique results for each tool.

### Unique and Shared References Between Tools

Each tool retrieved unique relevant citations in this study (see Figure 4). PubMed had the most unique, relevant results, with 31. Claude did best among LLMs, producing 21 real, relevant citations not retrieved by other tools.

Each tool also shared citations with others. Perplexity produced the most citations in common with other tools. Figure 5 depicts references shared by and unique to each tool.

**Figure 5:**
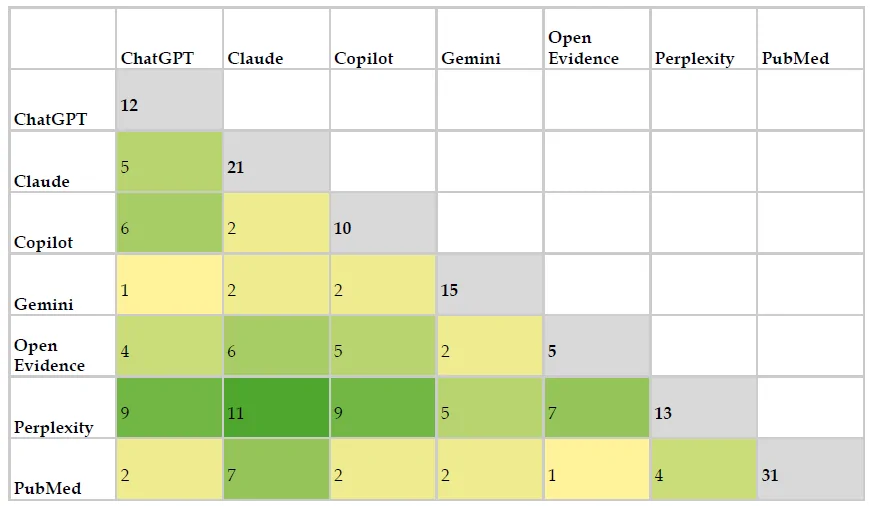
Real and relevant results unique to (gray boxes) and shared between (intersecting boxes) tools. Darker background indicates more shared citations.

## DISCUSSION

### Hallucinations

Based on our study, hallucination remains an issue for general AI tools providing article citations. Every tool except OpenEvidence returned hallucinated citations; OpenEvidence has not revealed source data, but is reported to have trained on collections of medical literature [22], and it is possible the tool accesses existing search features of these collections rather than using its LLM to generate citations. All other chatbots produced fake citations, usually generating hallucinated citations beginning with the first prompt response. Rate of hallucination was generally consistent across response iterations for each tool, suggesting that tools do not hallucinate more/less after multiple repeated prompts, at least for short conversations.

According to the Hallucination Leaderboard, hallucination rates of models used for this study likely ranged from 2–10% [23], but this is incomplete. OpenAI’s GPT-4o-Mini, some version of which most prompters used in this study, has a reported hallucination rate of 1.7%, but hallucinated 81% of citations we received from it. This may be because Hallucination Leaderboard’s reported rates reflect ideal circumstances, asking models to summarize documents and using a temperature of 0 (i.e., models were less likely to be “creative”). Since literature searches are not “ideal circumstances” for chatbots, and creating a hallucination-free LLM may be impossible [24], we suspect the problem of hallucinated citations will continue although newer models have lower reported hallucination rates.

### Relevance

Relevance is seen as a strength for these tools, and in our study they did mostly provide citations relevant to our topics.

The fact that AI tool results are usually relevant to prompts is likely important to users, who prefer not having to sort through irrelevant citations [25]. With most tools, topic accuracy is tempered by hallucinations, which uniformly seem relevant based on title but are useless for legitimate research. Advertising in chatbot responses is a developing field with potential to further cloud usefulness of prompt results [26–28]. The AI tools in our study still performed better in terms of relevance (for real results) than our simple PubMed searches, suggesting AI may be especially appealing to those non-expert searchers most likely to rely on basic searches in PubMed or other databases.

However, AI tools showed low return rate of real articles per prompt compared to PubMed searches, since they provided few results at once (averaging approximately 5 results per prompt iteration in this study, including hallucinations and duplications) and often hallucinated. Fewer results to review saves time for users with specific, limited information needs, but could make in-depth research more challenging since it is difficult to get a sense of the range of articles on a topic when retrieving only 4–5 citations at a time. Relevant articles per search were much higher in PubMed than with LLMs, suggesting that even for tools with few or no hallucinations, thorough scholarly work would ultimately save little if any time with AI searches, since repeating prompts to ensure coverage of a topic may equal the time needed to look through database result sets for those that are relevant.

### Repetition

All AI tools had high rates of duplicate results. This is not necessarily a concern in principle, since retrieving the same articles with variations on search strategies is also common in database and web searches. However, repetition potentially has greater impact here than with traditional search methods, given the smaller number of results from a single prompt of an AI tool. If each prompt retrieves only 5 citations, the potential for new useful results is lower than in a database or web search where dozens to hundreds of citations might result with each query. Frequent duplication over multiple small results sets may cause searchers to prematurely conclude that no additional relevant articles exist.

Despite attempting to cue tools by requesting “additional” articles in each follow-up prompt, we saw minimal evidence of new citations being preferentially returned in later iterations of prompts, though more varied prompting might have shown different results.

### Consistency

We observed low consistency of results between multiple users of AI tools, meaning a low likelihood of search replicability. While this may not concern an individual who is simply looking for an article on a topic, it could potentially hamper a shared, more complete understanding of the literature. If several people searching for information on the same subject with the same search terms see completely different articles, they could reach different conclusions by relying on AI tools. It is possible the tools would have shown more consistency if asked to summarize the literature on our topics rather than cite sources, but for researchers interested in going deeper than an AI overview, lack of consistency is a concern. Also concerning is AI tools’ lack of transparency: we have no way of knowing why any researcher saw the specific articles presented to them rather than those shown to someone else, meaning anything from random chance to stored information from previous prompts might influence which articles a researcher discovers.

Although our study took the viewpoint of an individual seeking literature on a simple topic, others have examined potential use of LLMs in various components of evidence synthesis projects [1,4,9,17]. The speed of AI chatbots could be valuable in these instances, where extensive work is required to locate and evaluate articles. However, reproducibility is a key component of systematic reviews [29], and the low consistency of citations produced by the LLMs we tested suggests they are currently inappropriate replacements for traditional databases such as PubMed. Systematic review teams should be advised to use LLMs only as a supplementary tool, perhaps useful for brainstorming search terms or phrasing queries, rather than for literature searching.

### Unique Results in Tools

Since each tool produced some real, relevant citations not provided by the others, a possible conclusion is that thorough, extensive literature searches might benefit from prompting even those AI platforms which did not perform well at this task by our measures. However, this is balanced by the time it took us to input the multiple prompts and remove hallucinated, duplicate and irrelevant results. It is also likely that sophisticated database and web searches such as those already employed by review teams would in fact discover those citations not retrieved with our simple PubMed searches.

### Potential Implications for Information Seekers

In academic settings, librarians have long promoted search skills training to students as time-saving, arguing that the more efficiently learners can locate articles relevant to their assignments, the more time they have for other components of their studies. Based on this metric and for this type of information need, it is difficult to frame an argument against the use of AI tools that is likely to resonate with students, since chatbots quickly provided relevant articles without need for advanced search skills. If an information seeker only wants a few articles on a topic to complete an assignment, an AI with minimal tendency to hallucinate may perform adequately.

However, for any in-depth information need, whether a systematic review project, historical retrospective, or simply a desire to understand the full scope of a topic, even low-hallucination AI tools demonstrated serious shortcomings due to their small results sets, lack of consistency, and tendency to repeat citations even when prompted for new ones.

Unlike PubMed and other scholarly databases, where the number of results for different searches can vary widely and is in part based on the amount of published literature for a topic, AI tools showed little variation in number of results for each prompt or topic. While more study is needed, we speculate that smaller retrieval from AI tools may promote a “flattened” perception of scholarly literature, suggesting an equal likelihood of retrieving a small number of relevant articles on any topic with a single request. Combined with the likelihood of seeing different articles than others investigating the same subject, even AI tools that do not hallucinate may contribute to a shallow and individualized understanding of a topic rather than a deeper, broader view that is more representative of the scholarly work.

At the same time, a cursory understanding is often preferable to no understanding, and chat tools’ ease of use enables quick acquisition of a few articles on a topic that may provide a basic overview. If these tools can interpret individual queries in the style most comfortable to each user and demonstrate an acceptable level of accuracy in returning an article, they may provide value to a researcher with a very basic need.

### Limitations

Study data was collected in August and September 2024. Since then, most of the LLM chatbots tested have released updated models with lower reported hallucination rates. Our study provides a broadly defined baseline to evaluate new models, not an assessment of current performance.

We considered only two questions, similarly worded. Different question types or more sophisticated prompt engineering could have produced results with fewer hallucinations and/or greater retrieval of new articles in later iterations. However, while both questions were medical in nature, one was clinically focused, and one educationally focused; we did not see meaningful differences in hallucination or duplication rates between them. We believe different question types will likely not lead to significant differences in hallucinated citations, duplication, or consistency of results.

Generalizability of data is limited by inconsistent numbers of prompters for each tool/prompt and inconsistent versions of tools. A more standardized dataset might have yielded different results and interpretations.

Finally, although the ability to translate and thereby facilitate sharing of information between languages is a potential strength of LLM tools, we considered prompts and results solely in English; findings of literature-searching projects in other languages might differ.

## CONCLUSIONS

Future research will undoubtedly continue to assess performance of AI tools in literature searching. Other research areas include the impact of AI tools on medical student information seeking, and the impact of prompt engineering on AI responses to literature requests (i.e., whether more sophisticated prompting skills deliver better results). Relatedly, if chat tools are accepted as standard resources for medical students seeking articles, librarians will need to teach patrons to effectively prompt for best results the way we now teach effective database and web searching: further study will determine best practices for both literature-search prompting and for presenting these skills in educational sessions.

Our results suggest that AI tools are not presently a viable replacement for PubMed or other academic databases for use in any in-depth literature searches; however, tools with little evidence of hallucination (in this study, OpenEvidence and Perplexity AI) may be adequate for limited information needs or as a complement to database or web searches.

## Data Availability

Data associated with this article are available in the Open Science Framework at https://osf.io/5xpe4/.
